# Potential Roles of m6A and FTO in Synaptic Connectivity and Major Depressive Disorder

**DOI:** 10.3390/ijms24076220

**Published:** 2023-03-25

**Authors:** Haruka Mitsuhashi, Corina Nagy

**Affiliations:** 1 McGill Group for Suicide Studies, Douglas Mental Health University Institute, McGill University, Montreal, QC H4H 1R3, Canada; 2 Integrated Program in Neuroscience, McGill University, Montreal, QC H3A 0G4, Canada; 3 Department of Psychiatry, McGill University, Montreal, QC H3A 0G4, Canada

**Keywords:** N6-methyladenosine, epitranscriptomics, FTO, major depressive disorder

## Abstract

RNA modifications known as epitranscriptomics have emerged as a novel layer of transcriptomic regulation. Like the well-studied epigenetic modifications characterized in DNA and on histone-tails, they have been shown to regulate activity-dependent gene expression and play a vital role in shaping synaptic connections in response to external stimuli. Among the hundreds of known RNA modifications, N6-methyladenosine (m6A) is the most abundant mRNA modification in eukaryotes. Through recognition of its binding proteins, m6A can regulate various aspects of mRNA metabolism and is essential for maintaining higher brain functions. Indeed, m6A is highly enriched in synapses and is involved in neuronal plasticity, learning and memory, and adult neurogenesis. m6A can also respond to environmental stimuli, suggesting an important role in linking molecular and behavioral stress. This review summarizes key findings from fields related to major depressive disorder (MDD) including stress and learning and memory, which suggest that activity-dependent m6A changes may, directly and indirectly, contribute to synaptic connectivity changes underlying MDD. Furthermore, we will highlight the roles of m6A and FTO, a m6A eraser, in the context of depressive-like behaviors. Although we have only begun to explore m6A in the context of MDD and psychiatry, elucidating a link between m6A and MDD presents a novel molecular mechanism underlying MDD pathogenesis.

## 1. Introduction

Epitranscriptomics, also known as RNA modifications, refers to the study of post-transcriptional modifications of RNA molecules. Although chemical modifications of RNA have been described for a half-century, only recently with the advancement of technology have we started to elucidate their functions [1]. To date, over 170 post-transcriptional base modifications have been identified. In addition, RNA modifications have been characterized not only in abundant non-coding RNA, such as transfer RNA (tRNA), ribosomal RNA (rRNA), and small nuclear RNA (snRNAs), but also in messenger RNA (mRNA) [2]. The post-transcriptional modifications of mRNA, which include N6-methyladenosine (m6A), N1-methyladenosine (m1A), 5-methylcytidine (m5C), N7-methylguanosine (m7G), and N6,2-O-dimethyladenosine (m6Am) add a new layer to regulating mRNA metabolism and gene expression [2]. Among them, m6A is one of the most abundant modifications of the mRNA in eukaryotes and the best-studied modification so far [3]. Given its diverse roles in mRNA metabolism and gene regulation, altered m6A profiles have been linked to various illnesses, including cancers and psychiatric disorders [4].

Major depressive disorder (MDD) is a complex, multifactorial illness, constituting one of the most important societal burdens worldwide [5]. While the genetic heritability of MDD is estimated to be at around 37%, there is no single large-effect variant [6]. As such, MDD, similar to many other psychiatric disorders, is likely to result from the combination of many small-effect variants that influence gene expression and interact with factors, such as the environment [7]. There is evidence to suggest that MDD is associated with disrupted neuronal communication within and across regions of the brain associated with mood regulation and cognitive function [8]. Indeed, numerous human post-mortem brain studies have found decreases in the sizes of these critical brain regions that some researchers have attributed to changes in synaptic density and neuropil loss [9,10,11]. Decreased synaptic density has important implications for intercellular communication and proper circuit function [12,13]. Various biological systems, such as the hypothalamic pituitary adrenal (HPA) axis, inflammation, sex steroids, and neurotropic factors, can lead to long- or short-term changes in neuronal communication through their influence on synaptic outgrowth and dendritic spine density [14]. Of note, the above-mentioned biological systems are all directly influenced by stress exposures throughout life, which, in turn, is one of the leading risk factors for the development of MDD [15]. Over the years, researchers have come to identify the molecular interface between stress and biology as epigenetics [16]. These are chemical modifications to nucleic acids that act as gene regulators that can respond to stressful stimuli and environmental factors.

Indeed, numerous studies, in both mice and humans, have shown that stressful exposures result in long-lasting epigenetic changes via DNA methylation and histone tail modifications [17]. Likewise, m6A is an epitranscriptomic modification that can dynamically respond to internal and external stimuli, resulting in the fine-tuning of the levels of gene expression. Notably, m6A participates in critical neurobiological functions, including synaptic plasticity, neurogenesis, and learning and memory [18,19]. Furthermore, m6A profiles are known to be altered by acute and chronic stress, suggesting that m6A is a player in stress-related psychiatric disorders, including MDD [20,21]. Given the role of m6A in brain function and, particularly, in activity-dependent gene regulation, it follows that m6A may act as a key regulator in the stress response. Consequently, this may have important implications for understanding the molecular mechanisms of MDD.

Others have done an excellent job of reviewing the roles of m6A in brain plasticity, learning and memory [18,19], and neurodegenerative disorders [22,23]. In this review, we focus on the evidence that m6A mediates synaptic plasticity in the context of MDD, which in turn, may result in some of the behavioral and clinical outcomes related to the pathology. First, we outline what is known about m6A in the brain, then we explore data from in vitro assays to human experiments, to summarize the involvement of m6A modifications on the various biologically associated pathways to MDD and to highlight the more direct evidence for m6A regulation in MDD.

## 2. m6A in the Brain

**Figure 1:**
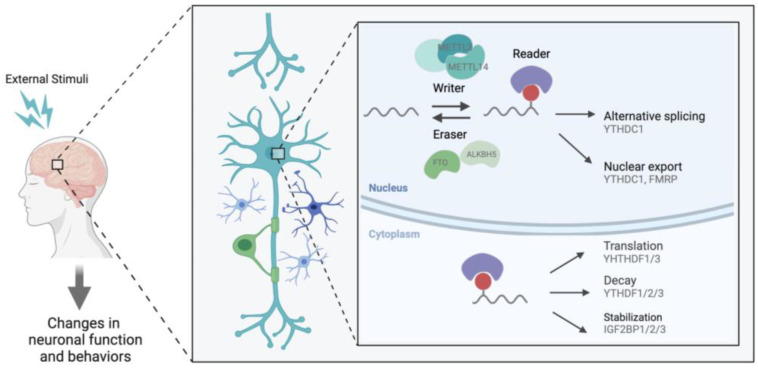
The suggested regulation of transcript by m6A in the nervous system. m6A is catalyzed by a writer complex consisting of METTL3, METTL14, WTAP, RBM15/15B, ZC3H13, VRMA, and HAKAI. m6A is demethylated by FTO or ALKBH5. m6A regulates the variety of mRNA metabolism in the nucleus and cytoplasm through recognition by readers, YTHDF1/2/3, YTHDC1,2, FMRP, HNRNPs, and IGFBP1/2/3. m6A has been shown to affect translation, degradation, splicing, and nuclear export and, therefore, could regulate the nervous system.

Methylation at the N6 position of adenosine is referred to as m6A. It is the most abundant RNA modification, with approximately 25% of mammalian messenger RNAs (mRNA) bearing the mark [3]. m6A is enriched in conserved regions, namely within the 3′ untranslated regions (UTRs) and near the stop codons of transcripts [24]. It has a known consensus motif RRACH (R represents A or G, and H represents A, C, or U) and, most importantly, it is dynamic and highly reversible [3,24,25]. m6A is known to be regulated at three complementary levels through proteins that act as writers, erasers, and readers [4,26] (Figure 1). Methylation is deposited by a multicomponent methyltransferase complex (“writers”) consisting of a core writer complex and an interacting complex. In the core complex, methyltransferase-like 3 (METTL3) is the catalytic component, methyltransferase-like 14 (METTL14) liaises with METTL3 to recognize the substrate, and WTAP guides METTL3/14 heterodimer [26]. The interacting complex contains the RNA binding motif protein 15/15B (RBM15/15B), vir-like m6A methyltransferase associated (VIRMA), zinc finger CCCH-type containing 13 (ZC3H13), and HAKAI which support the functioning and positioning of a writer complex. VIRMA interacts with WTAP and mediates selective methylation in the 3′UTR and near the stop codon [27]. RBM15/15B mediate the binding of a writer complex to the U enriched region on mRNA and recruit writer complexes to specific sites [28]. ZC3H13 mediates the nuclear localization of writer complexes [29,30]. Conversely, m6A is removed by demethylases (“erasers”), which include the fat mass and obesity-associated protein (FTO) and ALKBB homolog 5 (ALKBH5). For the mark to elicit its various effects, “reader” proteins must recognize and bind m6A, thereby regulating gene expression through diverse mechanisms such as mRNA stability, splicing, nuclear export, and translation efficiency [31]. Among the known m6A readers, the YTH family of proteins, with its conserved YTH-domain, is the best studied. Each member of the YTH family is reported to have a unique function; for example, YTHDC1 is predominantly found in the nucleus and promotes exon inclusion by selectively recruiting pre-mRNA splicing factor SRSF3 [32]. Further, YTHDC1 facilitates the nuclear export of methylated transcripts by interacting with nuclear transport receptors [33]. On the other hand, YTHDC2 and YTHDF1/2/3 are found in the cytoplasm. YTHDC2 has been shown to facilitate translation by resolving secondary structures or to promote mRNA degradation by interacting with 5′-3′ exoribonuclease [34,35]. In contrast, YTHDF2 destabilizes the target transcripts by recruiting a deadenylase complex [36]. YTHDF1 and YTHDF3 modulate translation efficiency [37,38]. However, recent studies propose that YTHDF1/2/3 bind to the same target transcripts and act redundantly to influence mRNA degradation [39]. This may not be surprising given that the YTHDF family has high sequence homology, sharing close to 85% of their sequence across the family [40]. Other readers include the fragile X mental retardation protein (FMRP), heterogeneous nuclear ribonucleoproteins (HNRNPs), and insulin-like growth factor 2 mRNA-binding proteins (IGF2BPs). FMRP facilitates the nuclear export of the target transcripts through interactions with mRNA nuclear export factors or a selective mRNA export pathway mediated by chromosomal maintenance 1 (CRM1) [41]. HNRNPC and HNRNPG modulate pre-mRNA processing and pre-mRNA alternative splicing, respectively [42]. In addition, HNRNPA2B1 is known to regulate alternative splicing and primary microRNA processing [43]. IGF2BP1-3 stabilizes the target transcripts and promotes the storage of mRNA by recruiting stabilizers [44]. More studies are needed to better understand the selective binding of m6A readers to different m6A sites; nonetheless, these findings support diverse molecular outcomes driven by how the m6A is read, functionally altering the levels of transcripts present in the cell.

Interestingly, m6A is highly enriched in the brain, with more than 30% of the transcripts in the brain harboring this modification [45]. Indeed, m6A profiles in the human brain show functional enrichment in synaptic and neuronal pathways for genes harboring brain-specific m6A [46]. Additionally, m6A writers, erasers, and readers are widely expressed throughout the brain, supporting the argument that m6A plays a vital role in various aspects of brain functions.

Although cell type-specific m6A profiles in the brain have not yet been generated at large, there is evidence to support that the proteins related to m6A are expressed across all cell types, albeit more highly expressed in the neurons compared to the glial cells [45]. However, given that non-neuronal cell types amount for at least half of all the cells in the brain, the roles of m6A in non-neuronal cell types cannot be overlooked. Indeed, studies have demonstrated the dynamic regulatory role of m6A in the oligodendrocyte lineage—a subclass of glial cells, including both oligodendrocyte precursor cells—and mature oligodendrocytes [47]. Thousands of transcripts are differentially methylated between the two cell types, implying that m6A may underlie cell-specific functions in the brain [47,48]. Taken together, this suggests a fundamental role for m6A methylation in the process of cellular differentiation.

## 3. Activity-Dependent Role of m6A

### 3.1. m6A Localizes Transcripts to the Synapse

The delivery of select transcripts to distal compartments, such as axons and dendrites, is critical in neurons where thousands of transcripts are locally regulated, influencing the production of proteins that affect synaptic organization and transmission. Recent research has shown that m6A modifies mRNAs destined for subcellular transport, specifically those targeted to the synapse. m6A profiles from the synaptosomes isolated from mouse forebrains found that m6A transcripts are enriched for pathways associated with synaptic function [49]. Specifically, mRNAs from the synaptosomes with high m6A levels were significantly enriched for synaptic functions compared to mRNAs with low methylation, which were more associated with cellular metabolic processes [49]. Moreover, m6A writers, erasers, and readers were found at dendrites and adjacent to synapses, suggesting that subcellular modification or the recruitment of modified mRNAs may play a role in synaptic activity [50]. Accordingly, the m6A profiling of neuronal subcellular compartments revealed that thousands of m6A transcripts are enriched in dendrites and axons compared to cell bodies [51]. Likewise, it has been found that the m6A sites within the 3′UTR promote the localization of a subset of transcripts to dendrites and axons in cultured mouse hippocampal neurons [51]. Furthermore, deleting the m6A writer METTL3 resulted in the altered localization of hundreds of transcripts [51]. Notably, the neurite-depleted transcripts corresponded to protein-coding genes associated with synaptic function and structure and neuronal projections. In line with this, knocking down the m6A readers YTHDF1 or YTHDF3 in cultured hippocampal pyramidal neurons decreased the translation of the dendritically localized mRNA and resulted in impaired synaptic transmission and abnormal dendrite spite morphology [49]. Similarly, YTHDF1 depletion was shown to impair hippocampal synaptic transmission and long-term transmission with a reduction in dendritic spine density [52]. It appears that readers are required to maintain translational efficiency at the presynaptic terminal.

Another mechanism by which local translation is regulated is through m6A eraser function within the axon, the effects of which have been associated with axon guidance, growth, and regeneration [53,54]. The eraser FTO is highly expressed in axons compared to other m6A machinery and has been shown to demethylate the local axonal mRNA [53]. The axon-specific loss of function of FTO inhibited the demethylation of the growth-associated protein-43 (GAP-43), which is required for axonal elongation [53]. The inhibition of demethylation increased m6A levels and, in turn, inhibited the local translation of GAP-43. Together these data suggest that m6A contributes to axon elongation by regulating the local translation of GAP-43 [53]. Additionally, m6A regulates axonal guidance by facilitating mRNA translation in the spinal cord. For example, for spinal commissural axons to cross the midline the m6A reader YTHDF1 must be inhibited, resulting in the decrease of axon guidance receptor Robo3.1 [54].

Taken together, the current literature suggests that m6A participates in the localization of target transcripts within neurons. Moreover, those targeted transcripts include the precursors for synaptic structural proteins which are important for synaptic communication. This implies that m6A may be one of the mediators in synaptic communication.

### 3.2. m6A and Synaptic Plasticity from Learning and Memory Studies

Synaptic plasticity is an essential part of the mechanism of neuronal adaptation. There is growing evidence to suggest that mice with mutations of m6A machinery genes affect the learning and memory processes. For example, the deletion of the m6A writer METTL3 in the hippocampus shows normal synaptic transmission and short-term memory but deficits in long-term memory consolidation [55]. At the molecular levels, METTL3 promotes the translation efficacy of immediate early genes (IEGS), which are induced rapidly by experience-triggered neuronal activity and are necessary for long-term memory [55]. Consistent with this finding, m6A writer METTL14 is required for long-term memory formation and neuronal excitability [56].

Likewise, the downregulation of the m6A eraser FTO and, thus, the increase of global m6A methylation level has been linked to learning and memory. At the basal levels, memory formation causes a short-term reduction in the abundance of FTO, preferentially at the synapse [57]. Moreover, FTO knockdown in the hippocampus before learning and memory training in mice enhanced memory formation in the contextual fear conditioning task [57]. Similarly, FTO knockdown in the mouse prefrontal cortex led to enhanced memory consolidation [56]. In a like manner, the depletion of FTO led to the upregulation of synaptic plasticity-related transcripts after an auditory fear conditioning behavioural task [21]. Similar to fear memory, the loss of FTO impaired working memory but did not influence long-term memory [58].

Readers, such as YTHDF1, have been shown to mediate the effect of m6A in learning and memory, most likely through the regulation of translation efficiency. For example, deleting YTHDF1 resulted in learning and memory defects, impaired hippocampal synaptic transmission, and long-term potentiation by promoting the translation of neuronal transcripts [52]. Conversely, restoring YTHDF1 in the hippocampus of *Ythdf1*-knockout mice rescued the behavioural and synaptic defects [52]. This change was accompanied by a decreased abundance of transcripts related to synaptic plasticity, including glutamate receptors and calcium calmodulin-dependent kinase (CaMK2a) [52]. Altogether, these data suggest that altered FTO expression in response to external stimuli plays a role in learning and memory, influencing the transcript level of gene-associated synaptic plasticity.

### 3.3. m6A Regulates Pathways Implicated in Psychiatric Disorders

Synaptic communication, within and across brain regions, is critical for mood regulation and cognitive function and alterations to synaptic function are a key feature of psychiatric disorders, including MDD. Emerging evidence implicates m6A in the molecular mechanisms closely associated with synaptic connectivity and psychiatric disorders. Indeed, recent studies have shown altered m6A profiles in neurodegenerative and psychiatric disorders, including Alzheimer’s disease [59,60,61], Parkinson’s disease [62], Huntington’s disease [63], alcohol use disorder [64], and post-traumatic stress disorder (PTSD) [65] in mice and human post-mortem brains [22,23].

As described above, m6A regulation has an important influence on synaptic plasticity, which has a direct impact on learning and memory, a system highly impacted by depression [14,66]. Other important biological systems implicated in depression, such as neurogenesis, HPA axis, inflammatory response, and neurotropic factors, are also shown to be regulated by m6A and will be discussed below.

While adult hippocampal neurogenesis (AHN) remains a controversial topic in humans, some studies have nonetheless shown an effect of AHN on psychiatric disorders with evidence that suggests m6A plays a role. The m6A eraser FTO is highly expressed in adult neural stem cells (aNSCs) and its loss reduces proliferation and neuronal differentiation in mice [58,67,68]. These changes are accompanied by an alteration in the methylation status of genes related to the brain-derived neurotrophic factor (BDNF) signaling pathway, which is known to promote the proliferation and differentiation of NSCs [67]. Another study showed the loss of FTO led to the disrupted precursor BDNF and mature BDNF [69]. BDNF is highly crucial for synaptic neuropil outgrowth, suggesting that m6A could be indirectly mediating synaptic deficits. Another study demonstrated that the *METTL3*-mediated m6A regulates neurogenesis and neuronal development by modulating the expression of histone methyltransferase Ezh2 [70]. METTL3 depletion inhibits the proliferation and cell cycle progression of aNSCs, with lineage commitment more toward glia during differentiation in vitro. Moreover, m6A is uniquely tagged under either proliferation or differentiation stages, suggesting that m6A may correlate with its functions in aNSCs and regulates neurogenesis at a normal state. Given the roles of m6A in adult neurogenesis, m6A may contribute to the development of MDD by regulating adult neurogenesis.

Dysregulation of the immune and inflammatory response, both peripherally and centrally, is commonly found across psychiatric disorders. Several studies have identified links between m6A and the inflammatory responses of microglia and macrophage, particularly in the polarization toward different phenotypes and inflammation: for example, genes related to microglia phenotypes. Pro-inflammatory-like M1-like, anti-inflammatory M2-like, and unstimulated M0-like are represented by state-specific methylation patterns, suggesting a role for m6A in the pro- and anti-inflammatory responses of the brain [71]. In macrophages, FTO knockdown led to changes in the gene expression of transcription factors essential for macrophage polarization and inhibited the nuclear factor-kappa B (NFkB) signaling pathway, thereby regulating macrophage activation [72]. In another study, METTL3 knockdown was shown to inhibit M1 polarization but enhance M2 polarization [73]. Similarly, YTHDF2 was shown to participate in the inflammatory response of macrophages by stabilizing the expression of inflammatory-related transcription factors and activating the MAPK and NFkB signaling pathways [74]. Although how m6A mediates inflammatory responses in the context of MDD is still unknown, m6A has been found to mediate the inflammatory response in the context of brain diseases such as brain stroke. Using an ischemic stroke model in mice, researchers identified an increase in m6A methylation in pathways vital to the inflammatory response, including tumour necrosis factor (TNF), Toll-like receptors (TLR), and NFkB [75].

## 4. Direct Evidence of the Involvement of m6A Regulation in MDD

As described above, a number of the systems that are dysregulated in MDD have been found to be mediated through m6A, but only a few studies have directly investigated the effects of m6A on depression and depressive-like behaviors. To date, many of these studies focused on an m6A eraser, FTO, due to its role in obesity and the strong link of metabolic disorders with depression [76,77]. Indeed, accumulating evidence shows the dysregulation of FTO in depressive pathology.

### 4.1. Potential Roles of m6A in Stress Response

The stress response is tightly regulated by the HPA axis, and it is responsible for translating stress-induced stimuli into hormonal signaling. Repeated stress can lead to increased HPA axis activity, increased levels of glucocorticoids, and disrupted negative feedback regulation, which are the hallmarks of MDD. Several studies have investigated the roles of m6A in response to stress. Acute stress was found to alter gene-expression levels of the erasers FTO and ALKBH5, which in turn regulated global m6A levels in the mice’s brains in a region-specific manner, with the global m6A levels increased in the amygdala and decreased in the prefrontal cortex in the mice [21]. In addition, injecting the glucocorticoid corticosterone mimics the acute stress effect and decreased erasers and increased global m6A levels, indicating that m6A may mediate stress response through the HPA axis [21]. However, in three separate mouse models of depression, changes in the level of erasers were not found in the prefrontal cortex or amygdala. Instead, FTO was consistently downregulated in the hippocampus of all three animal models and individuals with MDD [78]. In another study, chronic unpredictable stress (CUS) decreased global m6A levels in the hippocampus, which is opposite to previous findings [20]. Nonetheless, m6A profiling of the CUS model of the hippocampus revealed that genes with less methylation are enriched for neurotrophins and MAPK signaling pathways, both of which are dysregulated in MDD. Abdominal injections of the stress hormone corticosterone decreased global m6A levels in the peripheral blood in mice [21]. Similarly, the administration of synthetic stress hormones to individuals with MDD disrupted the global m6A levels in the peripheral blood [21]. In line with this, the deletion of FTO was shown to increase corticosterone levels in plasma, albeit the study was conducted at baseline levels [58].

Taking advantage of publicly available datasets, a recent study explored the gene expression level of m6A regulators in depression and depressive-like behaviors [79]. Although the expression patterns of m6A regulators after stress treatment or in individuals with MDD were inconsistent across studies, overall, the results suggested expression patterns were region and context-specific [20,21,78,79]. Altogether, rapidly modifiable methylation patterns as a function of stress or the introduction of synthetic stress hormones are in line with the dynamic regulation of transcripts to stress via m6A. However, more work is required to understand the context and region-specific effects.

### 4.2. KO Models Show Depressive-like/Anxiety-like Behaviors

To specifically investigate the impact of m6A on depressive- and anxiety-like behaviors, several studies tested the effects of manipulating m6A regulators in order to observe subsequent behavioral outcomes (Table 1). A study by Liu et al. showed that the suppression of FTO in the hippocampus increases depressive-like behaviors but not anxiety-like behaviors, while the overexpression of FTO led to an antidepressant-like effect [78]. Interestingly, *FTO* overexpression in mice resulted in hypomethylated genes enriched in pathways associated with synaptic organization and the regulation of neurotransmitters [78]. These pathways targeted by FTO were previously reported in depression and antidepressant-effects, suggesting that hippocampal FTO mediates, at least in part, depressive-like behaviors through cell-communication pathways. Consistent with this study, the downregulation of FTO in the anterior cingulate cortex led to depressive-like and anxiety-like behaviors through the changes in several key components of the BDNF pathway [80]. However, in contrast to these findings, *FTO*-knockout or *Mettl3*-knockout in the dorsal and ventral hippocampus did not lead to significant changes in anxiety-like behaviors but increases in fear memory [21]. Moreover, the global knockout of FTO resulted in reduced anxiety-like and depressive-like behaviors. However, FTO-knockout mice were too vulnerable and unable to tolerate CUMS [69]. Overall, the effect of FTO seems to depend on the stress paradigms, brain regions, and behavioral tests. However, these studies point toward the role of FTO as a key mediator of depressive-like behaviors.

Given the roles of m6A in depressive-like behaviors, it thus makes sense that m6A would play a role in antidepressant response. Indeed, a recent study investigated the potential role of m6A in response to antidepressant treatment. Peng-Fei et al. showed that FTO mediates tricyclic antidepressant (TCA) response in the ventral tegmental area (VTA) of mice. *FTO* knockout in the VTA increased stress vulnerability and abolished antidepressant activity, while FTO overexpression increased the activity of TCAs [81]. Moreover, overexpression of FTO in the VTA decreased the expression of stress-related neuropeptides in a chronic social defeat stress mice model (CSDS). However, it is important to note that the levels of FTO in response to antidepressants are region-specific. Moreover, different classes of antidepressants show different effects on the levels of FTO. Application of imipramine or amitriptyline, which are tricyclic antidepressants (TCAs), increased the expression of FTO, whereas fluoxetine, which is a selective serotonin reuptake inhibitor (SSRIs), decreased FTO in mouse neuroblastoma 2A cells. This is not overly surprising since each class, while influencing the levels of serotonin in the brain, nonetheless has different mechanisms of action.

### 4.3. Mutation in FTO and ALKBH5 Are Linked to MDD

Genome-wide association studies have linked variants in m6A-related genes to the risk of developing MDD and other psychiatric disorders [82,83]. Given that FTO is widely associated with obesity, and changes in appetite and weight are one of the common symptoms in MDD, FTO has been a novel candidate for these studies [76]. Early studies from Samman et al. investigated the association between the rs9939609 FTO risk variant and depression using four cohorts. Although the results were not replicated across all cohorts, the overall results from a meta-analysis showed that this SNP lowered the risk of depression [84]. Given the diversity and heterogeneity of depressive symptoms, researchers attempted to associate the different depressive subtypes with FTO [85]. In this study, MDD was separated into three categories: severe typical, moderate severity, and severe atypical, where the typical and atypical terms are mainly distinguished by the direction of the change in appetite, weight, and sleep [85]. In contrast to Samman et al., a positive relationship was identified between the FTO rs9939609 risk variant and depression in an atypical subtype of depression. Interestingly, a later meta-analysis was able to replicate these results [86]. Likewise, ALKBH5, another m6A eraser, was associated with depression in the Chinese Han cohort; however, these studies have yet to be replicated with larger sample sizes [87]. In other psychiatric disorders, the genetic variation of FTO was also shown to contribute to the risk of other psychiatric disorders, including Alzheimer’s disease [88,89].

## 5. Summary

**Figure 2:**
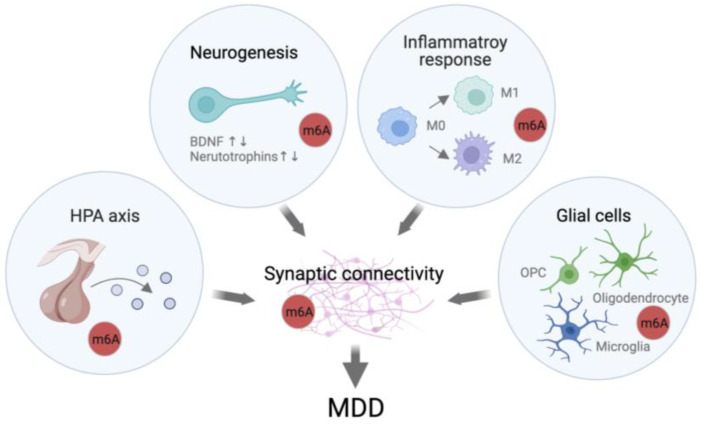
Model for the modulation of synaptic plasticity by m6A resulting in depressive phenotypes. Current literature points toward the role of m6A in biological systems, including the HPA axis, neurogenesis, inflammatory response, and glial functions, which have been implicated in depressive-like behaviors and MDD, suggesting a potential role of m6A in MDD.

m6A participates in a number of diverse functions in the adult brain that underlie neuronal activity and behavior (Figure 2). It is highly expressed at synapses and the dysregulation of m6A can disrupt synaptic connectivity and result in morphological changes. Studies from learning and memory and stress imply that activity-dependent m6A gene regulation can translate environmental stimuli into neuronal activity and behavior. In addition, m6A has been shown to participate in the inflammatory response, adult neurogenesis, expression of neurotropic factors, and the HPA axis, all of which have been strongly associated with MDD pathology. Taken together, m6A is highly responsive to stress, and is involved in stress response and adaptation. Of great interest, the current literature highlights the importance of FTO as a potential mediator in depressive-like behaviors.

Although recent literature points to the dysregulation of m6A in MDD and psychiatric disorders, there is a clear need for continued work in this field as we are only beginning to scratch the tip of the iceberg. For example, recent studies in MDD suggest that males and females respond differently to stress at multiple molecular levels. Therefore, studying the sex-specific regulation of m6A in the context of stress could identify the underlying mechanism engaged in the response to stressful events. Future studies using human postmortem brains could help us better understand the roles of m6A in MDD. Moreover, given the diverse cell types in the brain and their distinct roles in MDD, single-cell studies will be necessary to address the importance of m6A at a higher resolution. Future studies should also carefully investigate how m6A contributes to the response to antidepressant treatment, leveraging peripheral samples. Ultimately, these studies will help to identify novel pathways, mechanisms, and targets associated with MDD and move toward better interventions and treatments.

## Figures and Tables

**Table 1:** List of studies implicating m6A regulators in depressive-like behaviors.

Mice	Brain Region	Expression	Paradigm	Behavior	Citation
C57BL/6	HIPP	FTO Knockdown	-	Anxiety-like behaviors -	[21]
	HIPP	METTL3 Knockdown	-	Anxiety-like behaviors -	
C57BL/6	HIPP	FTO Knockdown	-	Anxiety-like behavior ↑	[58]
C57BL/6	Global	FTO Knockdown	-	Depressive-like behaviors ↓ Anxiety-like behaviors ↓	[69]
-	Global	FTO Knockdown	Chronic unpredictable mild stress	Unable to tolerate stress stimulation	
	Global	FTO Heterozygous (HZ)	Chronic unpredictable mild stress	Stress susceptible ↓	
C57BL/6	HIPP	FTO Knockdown	-	Depressive-like behaviors ↑ Anxiety-like behaviors-	[78]
	HIPP	FTO Knockout	-	Depressive-like behaviors ↑ Anxiety-like behaviors-	
	HIPP	FTO Overexpression	Chronic unpredictable mild stress	Depressive-like behaviors ↓	
C57BL/6	ACC	FTO Knockdown	Neuropathic pain	Depressive-like behaviors ↓ Anxiety-like behaviors ↓	[80]

## Data Availability

Not applicable.

## References

[1] Desrosiers R., Friderici K., Rottman F. (1974). Identification of methylated nucleosides in messenger RNA from Novikoff hepatoma cells. Proc. Natl. Acad. Sci. USA.

[2] Boccaletto P., Stefaniak F., Ray A., Cappannini A., Mukherjee S., Purta E., Kurkowska M., Shirvanizadeh N., Destefanis E., Groza P. (2022). MODOMICS: A database of RNA modification pathways. 2021 update. Nucleic Acids Res..

[3] Dominissini D., Moshitch-Moshkovitz S., Schwartz S., Salmon-Divon M., Ungar L., Osenberg S., Cesarkas K., Jacob-Hirsch J., Amariglio N., Kupiec M. (2012). Topology of the human and mouse m6A RNA methylomes revealed by m6A-seq. Nature.

[4] Yang C., Hu Y., Zhou B., Bao Y., Li Z., Gong C., Yang H., Wang S., Xiao Y. (2020). The role of m^6^A modification in physiology and disease. Cell Death Dis..

[5] Disease G.B.D., Injury I., Prevalence C. (2018). Global, regional, and national incidence, prevalence, and years lived with disability for 354 diseases and injuries for 195 countries and territories, 1990–2017: A systematic analysis for the Global Burden of Disease Study 2017. Lancet.

[6] Sullivan P.F., Neale M.C., Kendler K.S. (2000). Genetic epidemiology of major depression: Review and meta-analysis. Am. J. Psychiatry.

[7] Flint J., Kendler K.S. (2014). The genetics of major depression. Neuron.

[8] Duman R.S., Aghajanian G.K. (2012). Synaptic dysfunction in depression: Potential therapeutic targets. Science.

[9] Drevets W.C. (2000). Functional anatomical abnormalities in limbic and prefrontal cortical structures in major depression. Prog. Brain Res..

[10] Stockmeier C.A., Mahajan G.J., Konick L.C., Overholser J.C., Jurjus G.J., Meltzer H.Y., Uylings H.B., Friedman L., Rajkowska G. (2004). Cellular changes in the postmortem hippocampus in major depression. Biol. Psychiatry.

[11] Rajkowska G., Miguel-Hidalgo J.J., Wei J., Dilley G., Pittman S.D., Meltzer H.Y., Overholser J.C., Roth B.L., Stockmeier C.A. (1999). Morphometric evidence for neuronal and glial prefrontal cell pathology in major depression. Biol. Psychiatry.

[12] Kang H.J., Voleti B., Hajszan T., Rajkowska G., Stockmeier C.A., Licznerski P., Lepack A., Majik M.S., Jeong L.S., Banasr M. (2012). Decreased expression of synapse-related genes and loss of synapses in major depressive disorder. Nat. Med..

[13] Kaiser R.H., Andrews-Hanna J.R., Wager T.D., Pizzagalli D.A. (2015). Large-Scale Network Dysfunction in Major Depressive Disorder: A Meta-analysis of Resting-State Functional Connectivity. JAMA Psychiatry.

[14] Duman R.S., Aghajanian G.K., Sanacora G., Krystal J.H. (2016). Synaptic plasticity and depression: New insights from stress and rapid-acting antidepressants. Nat. Med..

[15] Turecki G. (2014). The molecular bases of the suicidal brain. Nat. Rev. Neurosci..

[16] Meaney M.J. (2010). Epigenetics and the biological definition of gene x environment interactions. Child Dev..

[17] Bagot R.C., Labonte B., Pena C.J., Nestler E.J. (2014). Epigenetic signaling in psychiatric disorders: Stress and depression. Dialogues Clin. Neurosci..

[18] Livneh I., Moshitch-Moshkovitz S., Amariglio N., Rechavi G., Dominissini D. (2020). The m^6^A epitranscriptome: Transcriptome plasticity in brain development and function. Nat. Rev. Neurosci..

[19] Widagdo J., Wong J.J.L., Anggono V. (2022). The m6A-epitranscriptome in brain plasticity, learning and memory. Semin. Cell Dev. Biol..

[20] Huang R., Zhang Y., Bai Y., Han B., Ju M., Chen B., Yang L., Wang Y., Zhang H.H.H., Zhang H.H.H. (2020). N^6^-Methyladenosine Modification of Fatty Acid Amide Hydrolase Messenger RNA in Circular RNA STAG1-Regulated Astrocyte Dysfunction and Depressive-like Behaviors. Biol. Psychiatry.

[21] Engel M., Eggert C., Kaplick P.M., Eder M., Roh S., Tietze L., Namendorf C., Arloth J., Weber P., Rex-Haffner M. (2018). The Role of m^6^A/m-RNA Methylation in Stress Response Regulation. Neuron.

[22] Zhang N., Ding C., Zuo Y., Peng Y., Zuo L. (2022). N6-methyladenosine and Neurological Diseases. Mol. Neurobiol..

[23] Zhang R., Zhang Y., Guo F., Li S., Cui H. (2022). RNA N6-Methyladenosine Modifications and Its Roles in Alzheimer’s Disease. Front. Cell. Neurosci..

[24] Meyer K.D., Saletore Y., Zumbo P., Elemento O., Mason C.E., Jaffrey S.R. (2012). Comprehensive analysis of mRNA methylation reveals enrichment in 3′ UTRs and near stop codons. Cell.

[25] Linder B., Grozhik A.V., Olarerin-George A.O., Meydan C., Mason C.E., Jaffrey S.R. (2015). Single-nucleotide-resolution mapping of m6A and m6Am throughout the transcriptome. Nat. Methods.

[26] He P.C., He C. (2021). m^6^A RNA methylation: From mechanisms to therapeutic potential. EMBO J..

[27] Yue Y., Liu J., Cui X., Cao J., Luo G., Zhang Z., Cheng T., Gao M., Shu X., Ma H. (2018). VIRMA mediates preferential m^6^A mRNA methylation in 3′UTR and near stop codon and associates with alternative polyadenylation. Cell Discov..

[28] Patil D.P., Chen C.K., Pickering B.F., Chow A., Jackson C., Guttman M., Jaffrey S.R. (2016). m^6^A RNA methylation promotes XIST-mediated transcriptional repression. Nature.

[29] Knuckles P., Lence T., Haussmann I.U., Jacob D., Kreim N., Carl S.H., Masiello I., Hares T., Villasenor R., Hess D. (2018). Zc3h13/Flacc is required for adenosine methylation by bridging the mRNA-binding factor Rbm15/Spenito to the m^6^A machinery component Wtap/Fl(2)d. Genes Dev..

[30] Wen J., Lv R., Ma H., Shen H., He C., Wang J., Jiao F., Liu H., Yang P., Tan L. (2018). Zc3h13 Regulates Nuclear RNA m^6^A Methylation and Mouse Embryonic Stem Cell Self-Renewal. Mol. Cell.

[31] Fu Y., Dominissini D., Rechavi G., He C. (2014). Gene expression regulation mediated through reversible m^6^A RNA methylation. Nat. Rev. Genet..

[32] Xiao W., Adhikari S., Dahal U., Chen Y.S., Hao Y.J., Sun B.F., Sun H.Y., Li A., Ping X.L., Lai W.Y. (2016). Nuclear m^6^A Reader YTHDC1 Regulates mRNA Splicing. Mol. Cell.

[33] Roundtree I.A., Luo G.Z., Zhang Z., Wang X., Zhou T., Cui Y., Sha J., Huang X., Guerrero L., Xie P. (2017). YTHDC1 mediates nuclear export of N6-methyladenosine methylated mRNAs. eLife.

[34] Mao Y., Dong L., Liu X.M., Guo J., Ma H., Shen B., Qian S.B. (2019). m^6^A in mRNA coding regions promotes translation via the RNA helicase-containing YTHDC2. Nat. Commun..

[35] Kretschmer J., Rao H., Hackert P., Sloan K.E., Hobartner C., Bohnsack M.T. (2018). The m^6^A reader protein YTHDC2 interacts with the small ribosomal subunit and the 5′-3′ exoribonuclease XRN1. RNA.

[36] Du H., Zhao Y., He J., Zhang Y., Xi H., Liu M., Ma J., Wu L. (2016). YTHDF2 destabilizes m^6^A-containing RNA through direct recruitment of the CCR4-NOT deadenylase complex. Nat. Commun..

[37] Shi H., Wang X., Lu Z., Zhao B.S., Ma H., Hsu P.J., Liu C., He C. (2017). YTHDF3 facilitates translation and decay of *N*^6^-methyladenosine-modified RNA. Cell Res..

[38] Wang X., Zhao B.S., Roundtree I.A., Lu Z., Han D., Ma H., Weng X., Chen K., Shi H., He C. (2015). N6-methyladenosine modulates messenger RNA translation efficiency. Cell.

[39] Zaccara S., Jaffrey S.R. (2020). A Unified Model for the Function of YTHDF Proteins in Regulating m6A-Modified mRNA. Cell.

[40] Hazra D., Chapat C., Graille M. (2019). m^6^A mRNA Destiny: Chained to the rhYTHm by the YTH-Containing Proteins. Genes.

[41] Hsu P.J., Shi H., Zhu A.C., Lu Z., Miller N., Edens B.M., Ma Y.C., He C. (2019). The RNA-binding protein FMRP facilitates the nuclear export of *N*^6^-methyladenosine-containing mRNAs. J. Biol. Chem..

[42] Liu N., Dai Q., Zheng G., He C., Parisien M., Pan T. (2015). *N*^6^-methyladenosine-dependent RNA structural switches regulate RNA-protein interactions. Nature.

[43] Alarcon C.R., Goodarzi H., Lee H., Liu X., Tavazoie S., Tavazoie S.F. (2015). HNRNPA2B1 Is a Mediator of m^6^A-Dependent Nuclear RNA Processing Events. Cell.

[44] Huang H., Weng H., Sun W., Qin X., Shi H., Wu H., Zhao B.S., Mesquita A., Liu C., Yuan C.L. (2018). Recognition of RNA *N*^6^-methyladenosine by IGF2BP proteins enhances mRNA stability and translation. Nat. Cell Biol..

[45] Chang M., Lv H., Zhang W., Ma C., He X., Zhao S., Zhang Z.W., Zeng Y.X., Song S., Niu Y. (2017). Region-specific RNA m^6^A methylation represents a new layer of control in the gene regulatory network in the mouse brain. Open Biol..

[46] Xiong X., Hou L., Park Y.P., Molinie B., Gregory R.I., Kellis M., Consortium G.T., Gregory R.I., Kellis M. (2021). Genetic drivers of m^6^A methylation in human brain, lung, heart and muscle. Nat. Genet..

[47] Xu H., Dzhashiashvili Y., Shah A., Kunjamma R.B., Weng Y.L., Elbaz B., Fei Q., Jones J.S., Li Y.I., Zhuang X. (2020). m6A mRNA Methylation Is Essential for Oligodendrocyte Maturation and CNS Myelination. Neuron.

[48] Wu R., Li A., Sun B., Sun J.G., Zhang J., Zhang T., Chen Y., Xiao Y., Gao Y., Zhang Q. (2019). A novel m^6^A reader Prrc2a controls oligodendroglial specification and myelination. Cell Res..

[49] Merkurjev D., Hong W.T., Iida K., Oomoto I., Goldie B.J., Yamaguti H., Ohara T., Kawaguchi S.Y., Hirano T., Martin K.C. (2018). Synaptic *N*^6^-methyladenosine (m^6^A) epitranscriptome reveals functional partitioning of localized transcripts. Nat. Neurosci..

[50] Flamand M.N., Meyer K.D. (2022). m6A and YTHDF proteins contribute to the localization of select neuronal mRNAs. Nucleic Acids. Res..

[51] Madugalle S.U., Meyer K., Wang D.O., Bredy T.W. (2020). RNA *N*^6^-Methyladenosine and the Regulation of RNA Localization and Function in the Brain. Trends Neurosci..

[52] Shi H., Zhang X., Weng Y.L., Lu Z., Liu Y., Lu Z., Li J., Hao P., Zhang Y., Zhang F. (2018). m6A facilitates hippocampus-dependent learning and memory through YTHDF1. Nature.

[53] Yu J., Chen M., Huang H., Zhu J.J., Song H., Zhu J.J., Park J., Ji S.J. (2018). Dynamic m6A modification regulates local translation of mRNA in axons. Nucleic Acids Res..

[54] Yu J., She Y., Yang L., Zhuang M., Han P., Liu J., Lin X., Wang N., Chen M., Jiang C. (2021). The m 6 A Readers YTHDF1 and YTHDF2 Synergistically Control Cerebellar Parallel Fiber Growth by Regulating Local Translation of the Key Wnt5a Signaling Components in Axons. Adv. Sci..

[55] Zhang Z., Wang M., Xie D., Huang Z., Zhang L., Yang Y., Ma D., Li W., Zhou Q., Yang Y.G. (2018). METTL3-mediated *N*^6^-methyladenosine mRNA modification enhances long-term memory consolidation. Cell Res..

[56] Widagdo J., Zhao Q.Y., Kempen M.J., Tan M.C., Ratnu V.S., Wei W., Leighton L., Spadaro P.A., Edson J., Anggono V. (2016). Experience-Dependent Accumulation of N6-Methyladenosine in the Prefrontal Cortex Is Associated with Memory Processes in Mice. J. Neurosci..

[57] Walters B.J., Mercaldo V., Gillon C.J., Yip M., Neve R.L., Boyce F.M., Frankland P.W., Josselyn S.A. (2017). The Role of The RNA Demethylase FTO (Fat Mass and Obesity-Associated) and mRNA Methylation in Hippocampal Memory Formation. Neuropsychopharmacology.

[58] Spychalaid A., Rü U. (2019). FTO affects hippocampal function by regulation of BDNF processing. PLoS ONE.

[59] Han M., Liu Z., Xu Y., Liu X., Wang D., Li F., Wang Y., Bi J. (2020). Abnormality of m6A mRNA Methylation Is Involved in Alzheimer’s Disease. Front. Neurosci..

[60] Li H., Ren Y., Mao K., Hua F., Yang Y., Wei N., Yue C., Li D., Zhang H. (2018). FTO is involved in Alzheimer’s disease by targeting TSC1-mTOR-Tau signaling. Biochem. Biophys. Res. Commun..

[61] Shafik A.M., Zhang F., Guo Z., Dai Q., Pajdzik K., Li Y., Kang Y., Yao B., Wu H., He C. (2021). N6-methyladenosine dynamics in neurodevelopment and aging, and its potential role in Alzheimer’s disease. Genome Biol..

[62] Chen Y., Chen Y., Song Y. (2021). Regulatory mechanism of FTO in Parkinson’s disease cell model. FASEB J..

[63] Pupak A., Singh A., Sancho-Balsells A., Alcalá-Vida R., Espina M., Giralt A., Martí E., Ørom U.A.V., Ginés S., Brito V. (2022). Altered m6A RNA methylation contributes to hippocampal memory deficits in Huntington’s disease mice. Cell. Mol. Life Sci. CMLS.

[64] Liu Y., Zhang H. (2022). RNA m6A Modification Changes in Postmortem Nucleus Accumbens of Subjects with Alcohol Use Disorder: A Pilot Study. Genes.

[65] Reis A.L.M., Hammond J.M., Stevanovski I., Arnold J.C., McGregor I.S., Deveson I.W., Gururajan A. (2022). Sex-specific transcriptomic and epitranscriptomic signatures of PTSD-like fear acquisition. iScience.

[66] Takeuchi T., Duszkiewicz A.J., Morris R.G. (2014). The synaptic plasticity and memory hypothesis: Encoding, storage and persistence. Philos. Trans. R. Soc. B.

[67] Li L., Zang L., Zhang F., Chen J., Shen H., Shu L., Liang F., Feng C., Chen D., Tao H. (2017). Fat mass and obesity-associated (FTO) protein regulates adult neurogenesis. Hum. Mol. Genet..

[68] Cao Y., Zhuang Y., Chen J., Xu W., Shou Y., Huang X., Shu Q., Li X. (2020). Dynamic effects of Fto in regulating the proliferation and differentiation of adult neural stem cells of mice. Hum. Mol. Genet.

[69] Sun L., Ma L., Zhang H., Cao Y., Wang C., Hou N., Huang N., von Deneen K.M., Zhao C., Shi Y. (2019). FTO deficiency reduces anxiety- and depression-like behaviors in mice via alterations in gut microbiota. Theranostics.

[70] Chen J., Zhang Y.J.Y.C., Huang C., Shen H., Sun B., Cheng X., Zhang Y.J.Y.C., Yang Y.G.Y., Shu Q., Yang Y.G.Y. (2019). m6A Regulates Neurogenesis and Neuronal Development by Modulating Histone Methyltransferase Ezh2. Genom. Proteom. Bioinform..

[71] Li Q., Wen S., Ye W., Zhao S., Liu X. (2021). The potential roles of m^6^A modification in regulating the inflammatory response in microglia. J. Neuroinflammation.

[72] Gu X., Zhang Y., Li D., Cai H., Cai L., Xu Q. (2020). N6-methyladenosine demethylase FTO promotes M1 and M2 macrophage activation. Cell. Signal..

[73] Liu Y., Liu Z., Tang H., Shen Y., Gong Z., Xie N., Zhang X., Wang W., Kong W., Zhou Y. (2019). The *N*^6^-methyladenosine (m^6^A)-forming enzyme METTL3 facilitates M1 macrophage polarization through the methylation of STAT1 mRNA. Am. J. Physiol. Cell Physiol..

[74] Yu R., Li Q., Feng Z., Cai L., Xu Q. (2019). m6A Reader YTHDF2 Regulates LPS-Induced Inflammatory Response. Int. J. Mol. Sci..

[75] Chokkalla A.K., Mehta S.L., Kim T., Chelluboina B., Kim J., Vemuganti R. (2019). Transient Focal Ischemia Significantly Alters the m^6^A Epitranscriptomic Tagging of RNAs in the Brain. Stroke.

[76] Loos R.J., Bouchard C. (2008). FTO: The first gene contributing to common forms of human obesity. Obes. Rev..

[77] Luppino F.S., de Wit L.M., Bouvy P.F., Stijnen T., Cuijpers P., Penninx B.W., Zitman F.G. (2010). Overweight, obesity, and depression: A systematic review and meta-analysis of longitudinal studies. Arch. Gen. Psychiatry.

[78] Liu S., Xiu J., Zhu C., Meng K., Li C., Han R., Du T., Li L., Xu L., Liu R. (2021). Fat mass and obesity-associated protein regulates RNA methylation associated with depression-like behavior in mice. Nat. Commun..

[79] Joshi K., Wang D.O., Gururajan A. (2022). The m6A-methylome in major depression: A bioinformatic analysis of publicly available datasets. Psychiatry Res. Commun..

[80] Wang X.-L., Wei X., Yuan J.-J., Mao Y.-Y., Wang Z.-Y., Xing N., Gu H.-W., Lin C.-H., Wang W.-T., Zhang W. (2022). Downregulation of Fat Mass and Obesity-Related Protein in the Anterior Cingulate Cortex participates in Anxiety- and Depression-Like Behaviors Induced by Neuropathic Pain. Front. Cell. Neurosci..

[81] Wu P.-F.F., Han Q.-Q.Q., Chen F.-F.F., Shen T.-T.T., Li Y.-H.H., Cao Y., Chen J.-G.G., Wang F. (2021). Erasing m6A-dependent transcription signature of stress-sensitive genes triggers antidepressant actions. Neurobiol. Stress.

[82] Choudhry Z., Sengupta S.M., Grizenko N., Thakur G.A., Fortier M.E., Schmitz N., Joober R. (2013). Association between obesity-related gene FTO and ADHD. Obesity.

[83] Rivera M., Locke A.E., Corre T., Czamara D., Wolf C., Ching-Lopez A., Milaneschi Y., Kloiber S., Cohen-Woods S., Rucker J. (2017). Interaction between the FTO gene, body mass index and depression: Meta-analysis of 13701 individuals. Br. J. Psychiatry.

[84] Samaan Z., Anand S.S., Zhang X., Desai D., Rivera M., Pare G., Thabane L., Xie C., Gerstein H., Engert J.C. (2013). The protective effect of the obesity-associated rs9939609 A variant in fat mass- and obesity-associated gene on depression. Mol. Psychiatry.

[85] Milaneschi Y., Lamers F., Mbarek H., Hottenga J.J., Boomsma D.I., Penninx B.W.J.H. (2014). The effect of FTO rs9939609 on major depression differs across MDD subtypes. Mol. Psychiatry.

[86] Yao Y., Wen Y., Du T., Sun N., Deng H., Ryan J., Rao S. (2016). Meta-analysis indicates that SNP rs9939609 within FTO is not associated with major depressive disorder (MDD) in Asian population. J. Affect. Disord..

[87] Du T., Rao S., Wu L., Ye N., Liu Z., Hu H., Xiu J., Shen Y., Xu Q. (2015). An association study of the m6A genes with major depressive disorder in Chinese Han population. J. Affect. Disord..

[88] Reitz C., Tosto G., Mayeux R., Luchsinger J.A., Group N.-L.N.F.S., Alzheimer’s Disease Neuroimaging I. (2012). Genetic variants in the Fat and Obesity Associated (FTO) gene and risk of Alzheimer’s disease. PLoS ONE.

[89] Keller L., Xu W., Wang H.X., Winblad B., Fratiglioni L., Graff C. (2011). The obesity related gene, FTO, interacts with APOE, and is associated with Alzheimer’s disease risk: A prospective cohort study. J. Alzheimers Dis..

